# A Dynamical Model of Pitch Memory Provides an Improved Basis for Implied Harmony Estimation

**DOI:** 10.3389/fpsyg.2017.00666

**Published:** 2017-05-04

**Authors:** Ji Chul Kim

**Affiliations:** ^1^Department of Psychological Sciences, University of ConnecticutStorrs, CT, USA; ^2^Oscilloscape LLCEast Hartford, CT, USA

**Keywords:** implied harmony, tonal melody, automatic chord estimation, pitch memory, dynamical system, neural oscillation, gradient frequency neural network

## Abstract

Tonal melody can imply vertical harmony through a sequence of tones. Current methods for automatic chord estimation commonly use chroma-based features extracted from audio signals. However, the implied harmony of unaccompanied melodies can be difficult to estimate on the basis of chroma content in the presence of frequent nonchord tones. Here we present a novel approach to automatic chord estimation based on the human perception of pitch sequences. We use cohesion and inhibition between pitches in auditory short-term memory to differentiate chord tones and nonchord tones in tonal melodies. We model short-term pitch memory as a gradient frequency neural network, which is a biologically realistic model of auditory neural processing. The model is a dynamical system consisting of a network of tonotopically tuned nonlinear oscillators driven by audio signals. The oscillators interact with each other through nonlinear resonance and lateral inhibition, and the pattern of oscillatory traces emerging from the interactions is taken as a measure of pitch salience. We test the model with a collection of unaccompanied tonal melodies to evaluate it as a feature extractor for chord estimation. We show that chord tones are selectively enhanced in the response of the model, thereby increasing the accuracy of implied harmony estimation. We also find that, like other existing features for chord estimation, the performance of the model can be improved by using segmented input signals. We discuss possible ways to expand the present model into a full chord estimation system within the dynamical systems framework.

## Introduction

Melody is a succession of pitched sounds arranged to form a coherent musical pattern (Bingham, 1910; Apel, 1969). In Western tonal melodies, coherence is often achieved by organizing melodic tones to imply harmonic progressions. Although tones in a melody sound successively in time, they can convey the sense of harmony, which is a relationship among simultaneously sounding pitches, by arpeggiating a chord and connecting chord tones via nonchord tones such as passing tones and neighbor tones (Schenker, 1956; Thomson, 1999). Psychological studies have shown that implied harmony is an important feature of the perception and cognition of tonal melodies (Cuddy et al., 1981; Tan et al., 1981; Trainor and Trehub, 1994; Holleran et al., 1995; Povel and Jansen, 2002).

Automatic chord estimation is a classic research area in music informatics aimed at identifying a sequence of chords that best matches the harmonic progression of a given music signal. Current signal-based approaches commonly employ chroma-based features such as chromagram which carry information on the energy distribution across 12 pitch classes or chromas (Jiang et al., 2011; Cho and Bello, 2014). Thus, chord estimation using these features is based on the duration and intensity of tones without taking their temporal order into account, which is consistent with the prevalent view of tonality perception and key-finding mechanisms based on pitch-class distributions (Krumhansl, 1990; Krumhansl and Cuddy, 2010). Chroma distributions are expected to be a reliable basis for chord estimation when there are more chord tones than nonchord tones in the frame of analysis. This is generally the case for harmonized music with explicit chordal support but not necessarily for unaccompanied melodies with frequent nonchord tones. Indeed, nonchord tones are recognized as a common source of errors in automatic chord estimation (Pardo and Birmingham, 2002; Lee and Slaney, 2006).

Here we present a novel feature extractor for automatic chord estimation that selectively enhances chord tones over nonchord tones on the basis of human perception of pitch sequences. Instead of analyzing chroma distributions in the acoustic signal, we use a model of human short-term pitch memory to determine the relative perceptual salience of individual tones in the signal. Psychological experiments have shown that pitches within a whole-tone range inhibit each other so that short-term retention of a pitch deteriorates when it is followed by a pitch neighbor (Deutsch, 1972, 1973; Deutsch and Feroe, 1975). Also, it has been shown that the memory of a melodic interval based on a simple frequency ratio (e.g., the perfect fifth based on 3:2) is more stable than the memory of a melodic interval based on a more complex ratio (e.g., the tritone which is approximated by 45:32) (Schellenberg and Trehub, 1994, 1996a,b). These findings suggest that melodic steps (a semitone and a whole tone) and leaps (intervals greater than a whole tone) have distinct perceptual properties: A pitch is weakened when it is followed by a step, while it becomes more salient when it forms a consonant leap with another pitch. Therefore, the salience of melodic pitches is determined not only by their duration but also by their temporal order (Bharucha, 1984; Brown, 1988) since the latter determines the pattern of steps and leaps. The differentiation between chord tones and nonchord tones may arise from the pattern of cohesion and competition among melodic pitches in short-term auditory memory, such that salient pitches that cohere together are heard as chord tones whereas pitches suppressed by others serve as nonchord tones (Kim, 2011; Kim and Large, under revision).

In this paper, we test pitch interactions arising from the pattern of melodic steps and leaps as a basis for automatic chord estimation. To model the interaction of melodic pitches in auditory memory, we use a network of tonotopically tuned nonlinear oscillators. This is not an arbitrary choice of implementation. Rather, it is based on the observation that the two distinct types of pitch interaction discussed above—inhibition by pitch neighbors and coherence based on simple frequency relationships—correspond with the two characteristic behaviors of nonlinear systems: lateral inhibition and nonlinear resonance. The model, which is described below, is a dynamical system; it is run by numerically integrating a set of differential equations which specify the dynamics and interactions of its components. Therefore, it runs forward in time (i.e., it can potentially run in realtime) and does not involve any search procedures or optimization steps that require access to an entire time series. The model is driven by audio signals, and acoustic frequencies are transformed into a complex pattern of oscillations which we take as a measure of pitch salience. We test the model with unaccompanied tonal melodies and show that chord tones are selectively enhanced in the response of the model compared to the distribution of physical tone durations.

## General material and methods

### Model

We model short-term pitch memory with a network of tonotopically tuned nonlinear oscillators, which is known as a *gradient frequency neural network* (abbreviated as GrFNN and pronounced *griffin*; Large et al., 2010). Nonlinear oscillation is found in many parts of the auditory system, including critical oscillations in the cochlea (Camalet et al., 2000; Hudspeth et al., 2010) and mode-locked firing of auditory subcortical neurons (Large et al., 1998; Laudanski et al., 2010). We use a generic mathematical form of nonlinear oscillation, called the canonical model, which describes oscillatory activities with complex-valued state variables (Kim and Large, 2015). GrFNNs have been used successfully to model auditory neural processing (Lerud et al., 2014, 2015) as well as music cognition (Large et al., 2015, 2016).

**Figure 1:**
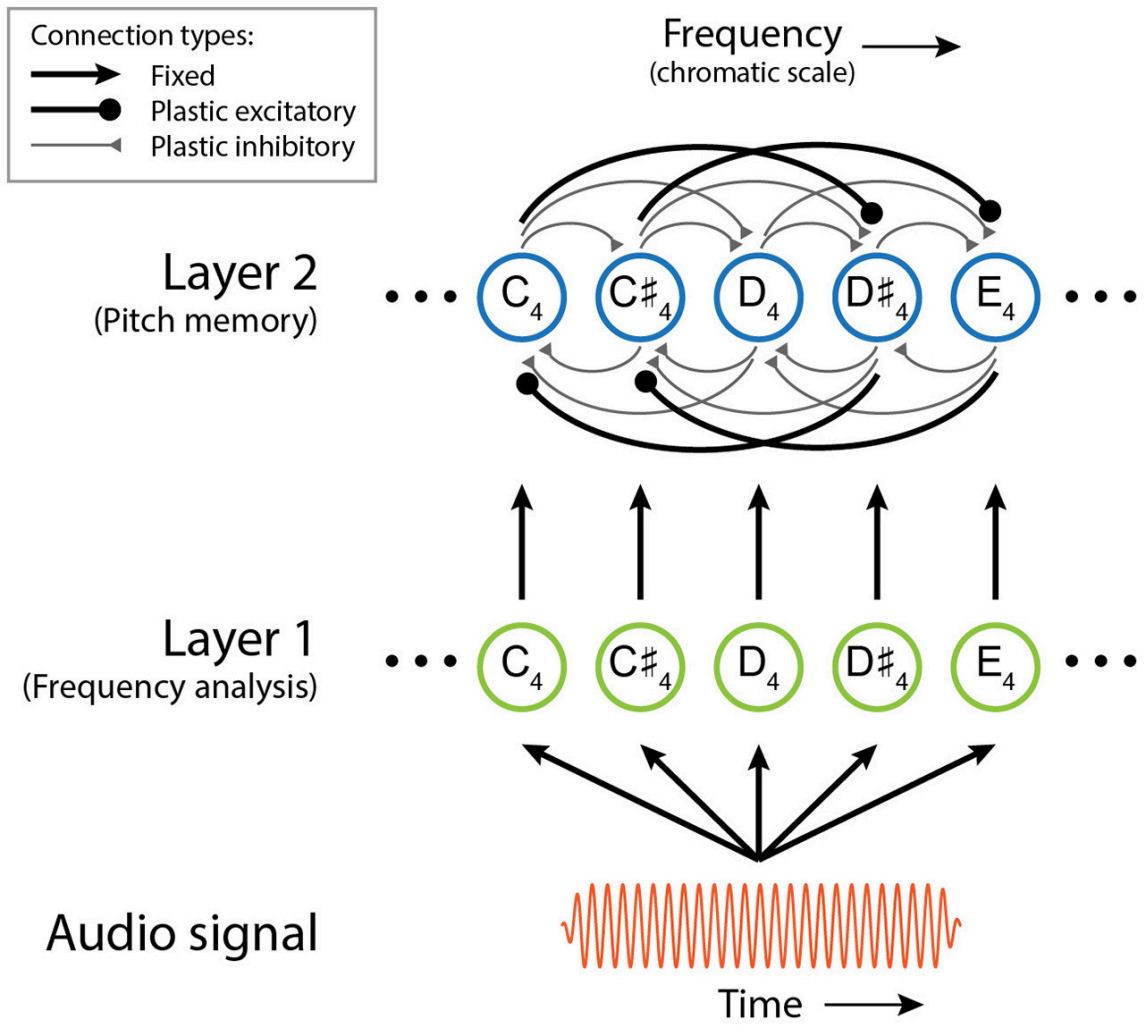
**Schematic of the dynamical model of short-term pitch memory**. The colors and line widths used for different connection types are only for visual distinction and do not indicate their relative strengths.

**Figure 2:**
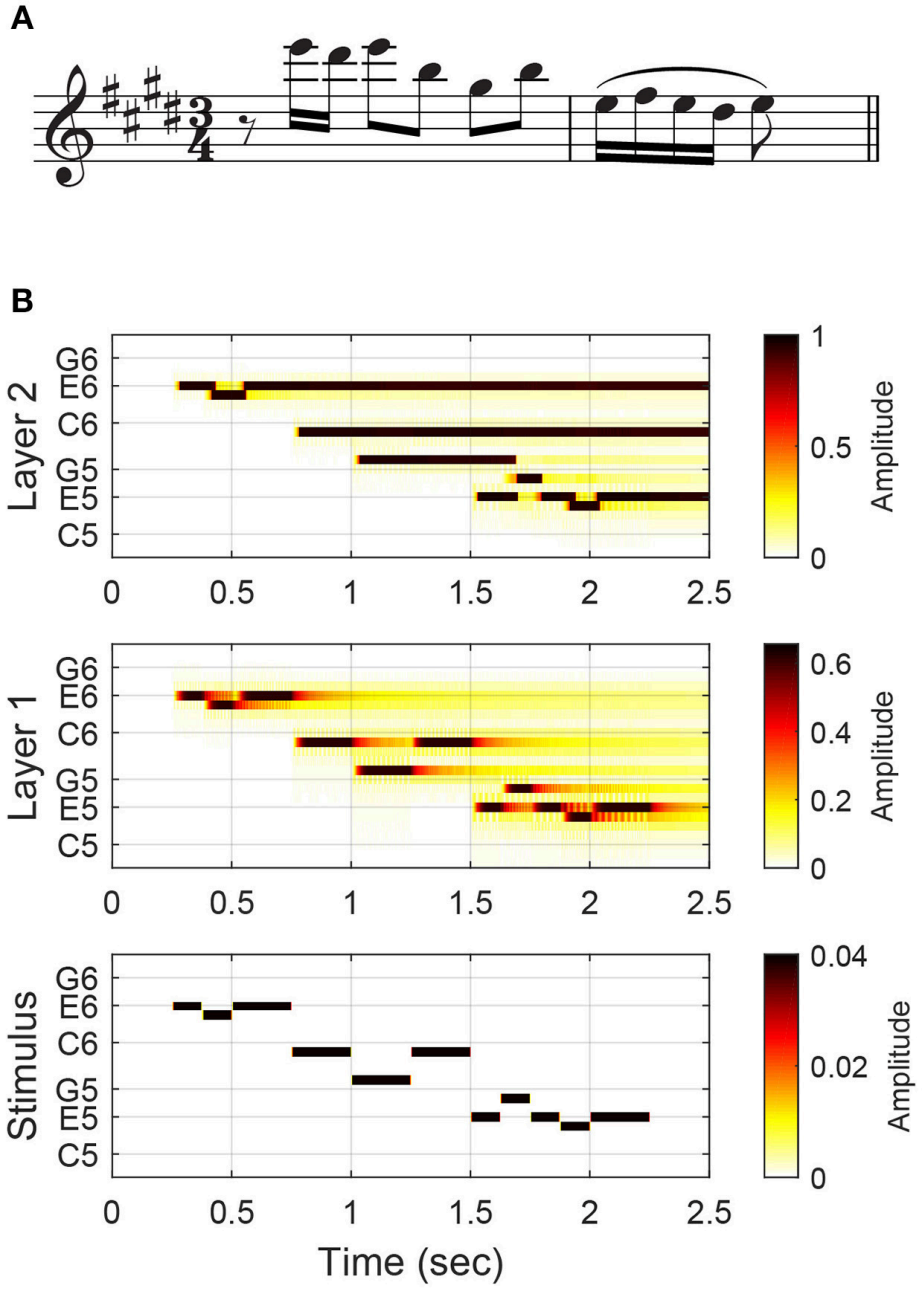
**The model's response to the opening of J. S. Bach's Violin Partita No. 3, BWV 1006, Prelude: (A)** the musical score and **(B)** the amplitudes of Layer 1 and Layer 2 oscillators and stimulus tones. The stimulus (an audio signal) is depicted in a piano-roll representation. High-amplitude oscillations in Layer 2 (depicted with dark colors) are considered active pitch traces in auditory memory.

Here we describe the structure and function of the short-term pitch memory model with an example. (The differential equations governing the dynamics of the model are given below, along with the parameter values used in this study, but understanding of the mathematical details is not required to comprehend the results and implications of this study.) The model consists of two layers of nonlinear oscillators tuned to a chromatic scale (Figure 1). Layer 1 is driven by an audio signal and performs frequency analysis. Figure 2 shows the response of the model to a passage composed by J. S. Bach for solo violin. Layer 1 oscillators resonate to different frequencies so that they separate out individual frequencies in the signal. The parameters for Layer 1 oscillators were chosen to capture the critical oscillations observed in the cochlea (see Equation 1 below for more details).

Layer 2 is a model of short-term pitch memory. High-amplitude oscillations above the on-threshold (see below) are considered active pitch traces that are salient in auditory memory. Layer 2 receives input from Layer 1 and includes internal pairwise connections between all oscillators (see Figure 1 and Equation 2 below). Through these connections, Layer 2 oscillators either inhibit or resonate with each other depending on their frequency relationships. Two oscillators inhibit each other if their natural frequencies are a semitone or a whole tone apart. So a Layer 2 oscillation is suppressed when its stimulus tone is followed by another tone within a whole-tone distance. For example, the memory trace for the second tone (D♯6) in the Bach melody is suppressed at the onset of the following tone (E6) which is a semitone apart (Figure 2B). When the natural frequencies are more than a whole tone apart, the oscillators resonate together by synchronizing in an integer ratio (called mode-locking). Nonlinear resonance is stronger for simpler frequency relationships such as 2:1 (an octave) and 3:2 (a perfect fifth) so that oscillations driven by a consonant leap last longer than oscillations for a dissonant leap. For example, the oscillatory traces at E6 and B5, which are a perfect fifth apart, are sustained long beyond the physical duration of the tones (Figure 2B). The parameters for Layer 2 oscillators were chosen so that they have thresholds for turning on and off which simulates the persistence and loss of memory traces.

The pairwise connections between Layer 2 oscillators are governed by a Hebbian learning rule (Equation 3). The plastic connections model short-term adaptation in the auditory system rather than long-term learning. The connections strengthen and weaken quickly depending on the current amplitude and frequency relationship of their source and target oscillators. When two Layer 2 oscillators in a simple frequency relationship have high amplitudes at the same time, the plastic connections between them quickly strengthen and let the oscillators reinforce each other through nonlinear resonance (i.e., mode-locking). When two oscillators within a whole-tone range are activated simultaneously, the connections between them grow quickly but they introduce lateral inhibition so that the oscillator with higher amplitude (typically the one currently driven by a stimulus tone) suppresses the other oscillator. The plastic connections decay quickly as either of the oscillators goes below the off-threshold.

Let us discuss how the pitch memory model can improve the estimation of implied harmony by selectively enhancing chord tones over nonchord tones. Bach's pieces for solo instruments, such as the passage shown in Figure 2A, are well known for creating an impression of vertical harmony out of a single unaccompanied line (Davis, 2006). The oscillatory patterns formed in Layer 2 show how this may be possible (Figure 2B). The first group of notes (E-D♯-E) leaves one oscillatory trace at E6, with the trace for the neighbor tone (D♯6) confined to the time of physical sounding due to lateral inhibition. The next three notes (B-G♯-B) form consonant leaps, so their traces prolong together without inhibiting each other (note that the trace at B5 is sustained through a temporal gap). The last five notes form a turn figure made of only steps, so only the trace for the last note (E5) is extended. At the end of the passage, the oscillations at E6, B5 and E5 remain active. Along with the trace at G♯5, which prolongs beyond the note duration before being suppressed by the following F♯5, the active oscillatory traces suggest that the melody implies an E-major harmony. It is possible to estimate the chord from note durations (the chord tones take up 81% of total notated duration), but chord tones are made more salient in the response of the model (the chord tones take up 92% of total trace duration, excluding prolongations past the offset of the last note). Below we take the length of oscillatory traces as a measure of pitch salience and test if it can serve as a better basis for chord estimation than note durations.

Equations (1–3) specify the time evolution of each component in the dynamical model. (The readers may skip the equations and proceed to the Material section.) Equation (1) describes the interaction of Layer 1 oscillators with an external signal.

(1) $$\begin{array}{r} \tau_{1}\frac{dz_{1i}}{dt}=z_{1i}\left(\alpha_{1}+\text{i}2\pi f_{i}+\beta_{11}|z_{1i}{|}^{2}+\frac{\epsilon_{1}\beta_{12}|z_{1i}{|}^{4}}{1-\epsilon_{1}|z_{1i}{|}^{2}}\right)+x\left(t\right), \end{array}$$

where *z*_1*i*_ is a complex-valued state variable specifying the amplitude and phase of the *i*th oscillator in Layer 1, *f*_*i*_ is its natural frequency, *x*(*t*) is a complex-valued external signal which can be obtained by applying the Hilbert transform to a real-valued audio signal, and the roman i is the imaginary unit. The parameters α_1_, β_11_, β_12_, and ϵ_1_ determine the intrinsic dynamics of the oscillators, and τ_1_ is the time constant (see Kim and Large, 2015, for an analysis of all intrinsic dynamics available in the canonical model). The parameter values used are α_1_ = 0, β_11_ = −0.1, β_12_ = −0.1, ϵ_1_ = 1, and τ_1_ = 0.0025 (this is the critical Hopf regime, known to underlie cochlear dynamics; see Kim and Large, 2015).

Equation (2) determines the dynamics of Layer 2 oscillators (*z*_2*i*_) which receive input from Layer 1 oscillators of identical natural frequencies (*z*_1*i*_) as well as from all other oscillators in Layer 2 (*z*_2*j*_).

(2) $$\begin{array}{l} \begin{array}{c} \tau_{2i}\frac{dz_{2i}}{dt}=z_{2i}\left(\alpha_{2}+\text{i}2\pi +\beta_{21}|z_{2i}{|}^{2}+\frac{\epsilon_{2}\beta_{22}|z_{2i}{|}^{4}}{1-\epsilon_{2}|z_{2i}{|}^{2}}\right)+c_{\text{aff}}z_{1i}\\ \;+\underset{j\neq i}{\sum }{\sqrt{\epsilon_{2}}}^{k_{ij}+m_{ij}-2}c_{ij}z_{2j}^{k_{ij}}{\bar{z}}_{2i}^{m_{ij}-1}, \end{array} \end{array}$$

where *c*_*ij*_ is a complex state variable for the plastic connection from the *j*th oscillator to the *i*th oscillator, and *c*_aff_ is the strength of afferent connections. *k*_*ij*_ and *m*_*ij*_ are integers that approximate the frequency ratio of the *i*th and *j*th oscillators (i.e., *k*_*ij*_ : *m*_*ij*_ ≈ *f*_*i*_ : *f*_*j*_), which corresponds to the ratio of mode-locking. The parameter values used are α_2_ = −1.6, β_21_ = 2.2, β_22_ = −0.1, ϵ_2_ = 1, τ_2*i*_ = 1/*f*_*i*_, and *c*_aff_ = 1.5 (this is the subcritical double limit cycle regime which exhibits hysteresis with different on- and off-thresholds; see Kim and Large, 2015).

The evolution of plastic connections between Layer 2 oscillators (*c*_*ij*_) is determined by a Hebbian learning rule,

(3) $$\begin{array}{r} \tau_{ij}\frac{dc_{ij}}{dt}=c_{ij}\left(\lambda_{ij}+\mu_{1ij}|c_{ij}{|}^{2}+\frac{\epsilon_{c}\mu_{2ij}|c_{ij}{|}^{4}}{1-\epsilon_{c}|c_{ij}{|}^{2}}\right)\\ +\;{\sqrt{\epsilon_{c}}}^{k_{ij}+m_{ij}-2}\kappa_{ij}z_{2i}^{m_{ij}}{\bar{z}}_{2j}^{k_{ij}}. \end{array}$$

Different parameter values were used depending on the interval between the natural frequencies of the source and target oscillators. For a semitone difference: λ_*ij*_ = −1, μ_1*ij*_ = 0, μ_2*ij*_ = −1 and κ_*ij*_ = −0.5 (inhibitory). For a whole tone difference: λ_*ij*_ = −1, μ_1*ij*_ = 0, μ_2*ij*_ = −1 and κ_*ij*_ = −1 (inhibitory). For a difference greater than a whole tone: λ_*ij*_ = −0.1, μ_1*ij*_ = 0, μ_2*ij*_ = −10000 and κ_*ij*_ = 0.02 (excitatory). For all three cases: ϵ_*c*_ = 1 and $\tau_{ij}=\frac{k_{ij}+m_{ij}}{k_{ij}f_{j}+m_{ij}f_{i}}$.

### Material

We tested the dynamical model with tonal melodies from seven Mozart piano sonatas (K. 279, K. 280, K. 281, K. 282, K. 283, K. 331, and K. 545). We took the top voice from the expositions of the first movements in sonata form. For K. 311, which is a theme and variations, the melody was taken from the theme. We selected these melodies because they are accompanied by mostly unambiguous chordal support in the left hand. We relied on both the melody and the accompaniment to annotate each note in the melody with the underlying chord and whether the note is a chord tone or a nonchord tone. The Mozart melodies include ample nonchord tones (593 nonchord tones out of 2,020 notes, comprising 29% of total notes) compared to other collections we considered (e.g., nonchord tones represent only 7% of the notes in the vocal part of Schumann's *Dichterliebe*). This makes the Mozart melodies good materials to test for the differentiation between chord tones and nonchord tones. We used the annotations (based on both the melody and the accompaniment) to evaluate the model's responses to the unaccompanied melodies. The annotations should not be considered as the only possible harmonic interpretations since the harmony implied by a melody (without accompaniment) could differ from the harmony of the accompaniment (Temperley, 2007). Also, it is common knowledge that the same melody can be harmonized in many different ways. These potential discrepancies, however, would only make the model's predictions less accurate. Thus, the tests reported below should be considered conservative tests.

For each Mozart melody, we created an audio signal made of pure tones (complex-valued sinusoids) that match the notated pitches and durations in the score. An amplitude envelope was applied to each stimulus tone, with sustained amplitude of 0.04 and linear ramps of 5 ms at the onset and the offset. The use of pure tones, instead of complex tones, is due to the limitation of Layer 1 in the current form. Layer 2 is a model of short-term pitch memory which takes oscillations at individual *pitches* as input. Layer 1, however, separates individual spectral components in the audio signal rather than extracting individual pitches (or fundamental frequencies) from them. Instead of incorporating pitch estimation into the model (which requires more than frequency analysis; see, e.g., de Cheveigné, 2006), here we use audio signals containing only pure tones for which pitches can be obtained by frequency analysis alone. Currently we are developing a GrFNN pitch estimator, and the future versions of the present model will include a pitch estimator and thus be able to handle signals containing complex sounds.

### Methods

For each stimulus signal, the model was run by numerically integrating Equations (1–3) using GrFNN Toolbox (Large et al., 2014), which is a software library for building and running GrFNN models. Before each integration, all oscillators and plastic connections in the model were set to random initial conditions with small amplitudes. The range of natural frequencies in the model was determined by the pitch range of the stimulus melody. The natural frequencies of the oscillators spanned from three semitones below the lowest note in the melody up to three semitones above the highest note. For stable fixed-step numerical integration, the sampling frequency was set to 20 times the highest natural frequency in the model.

The duration of oscillatory traces in Layer 2 was taken as a measure of pitch salience. Trace duration was defined as the length of time from the moment a Layer 2 oscillation jumps above the on-threshold until either the moment it drops below the off-threshold or the next note onset at the same pitch or the offset of the last note in the signal (or the last note in the chord span for Test 2), whichever occurs first. So if a trace is extended into another trace at the same pitch, the trace duration for the first tone is counted only up to the onset of the second tone. For the parameter values used in this study, the on- and off-thresholds were 0.89 and 0.50 respectively. Note duration was defined as the length of time for which the stimulus tone stays above 50% of its maximum amplitude.

## Test 1: trace prolongation for chord tones and nonchord tones

To test whether chord tones are selectively emphasized in the model's response, we compared the trace durations for chord tones and nonchord tones. Given the high probability of nonchord tones being followed by a step (Bharucha, 1996), we predicted that the oscillatory traces driven by nonchord tones would mostly end soon after the note offsets while the traces for chord tones would often prolong beyond the note durations. We tested this prediction by comparing the difference between trace duration and note duration (hereafter, *trace prolongation*) for chord tones and nonchord tones.

### Methods

The model was run for each of the Mozart melodies separately (see General Material and Methods above for details). For each note in the melodies (marked either as a chord tone or a nonchord tone), note duration, trace duration and trace prolongation (= trace duration − note duration) were determined. A *t*-test was performed to determine if chord tones and nonchord tones had significantly different trace prolongations.

### Results and discussion

**Figure 3:**
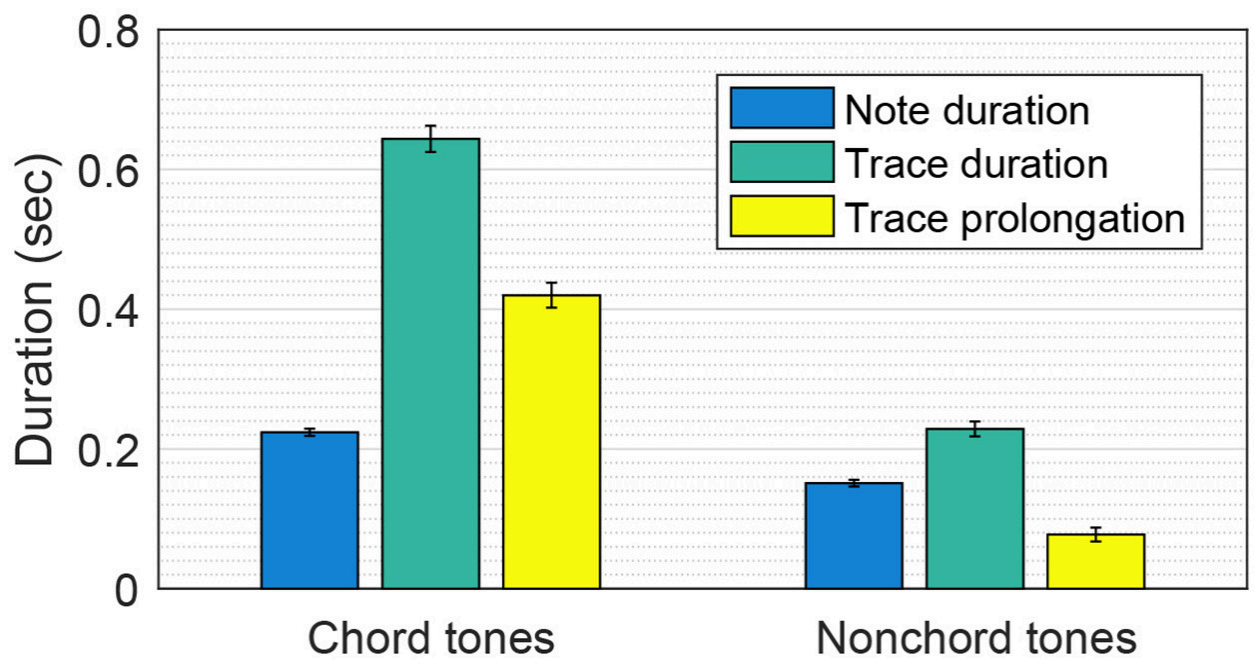
**Comparison of the trace prolongations for chord tones and nonchord tones in the Mozart melodies**. Mean note duration, mean trace duration and mean trace prolongation (i.e., trace duration − note duration) are shown. The error bars indicate standard errors.

The chord tones in the Mozart melodies had significantly longer trace prolongations than the nonchord tones [two-sample *t*-test: *t*(2, 018) = 12.07, *p* < 0.001]. The mean trace prolongations for chord tones and nonchord tones were 420 and 76 ms, respectively (see Figure 3). This means that the chord tones were more emphasized in the pitch memory model than in the note durations. The note durations for chord tones and nonchord tones were also significantly different [mean durations: 224 and 151 ms; *t*(2, 018) = 8.57, *p* < 0.001]. However, this difference does not explain the difference in trace prolongation because the trace prolongation for an isolated tone does not depend on the note duration, provided that the tone is long enough to activate an oscillatory trace (which is true for all notes in the Mozart melodies). Thus, longer trace prolongations for chord tones are attributed to the nonlinear interaction between oscillatory traces (i.e., inhibition and resonance) in conjunction with the fact that nonchord tones are followed by step more often (91% of the time in the Mozart melodies) than chord tones are (52%).

It is important to note that chord tones are selectively enhanced in the pitch memory model because of the regularities in the use of chord tones and nonchord tones in tonal music. A basic rule of counterpoint states that a nonchord tone (or a dissonance) must be resolved by step motion (Zarlino, 1558; Fux, 1725). The pitch traces for nonchord tones are prolonged to a lesser extent than the traces for chord tones because nonchord tones are mostly followed by a step whereas chord tones have no such restriction. If the opposite was true (i.e., chord tones were followed by a step while nonchord tones had no constraint), nonchord tones would be emphasized in the response of the model. Then, one could ask why chord tones and nonchord tones are used in certain ways, which is by no means limited to Western tonal music (Erickson, 1984; Thomson, 1999). It is reasonable to assume that the way melodic pitches interact in auditory memory has guided and constrained the way chord tones and nonchord tones are used in tonal music. The function of nonchord tones is to embellish chord tones without undermining their structural and perceptual prominence. Thus, one would want to limit the salience of nonchord tones while highlighting chord tones. Stepwise resolution of nonchord tones, which leads to the suppression of their pitch salience, may be viewed as a compositional practice evolved under the selective pressure by the principles of pitch organization in auditory memory.

## Test 2: trace durations within chord spans

**Figure 4:**
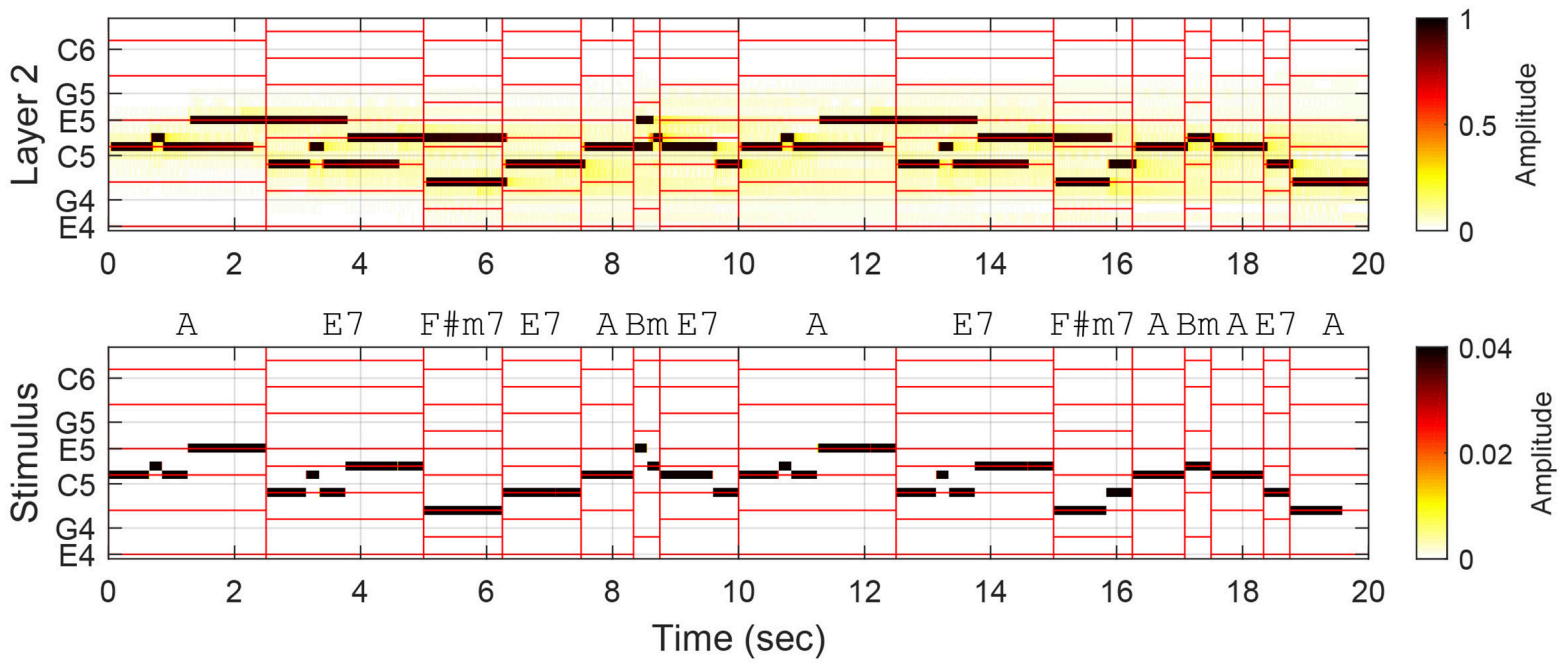
**Oscillatory traces formed in Layer 2 in response to the first two phrases (the first 15 chord spans) in Mozart Piano Sonata No. 11, K. 331, Theme**. Vertical red lines demarcate chord spans, and horizontal lines indicate the pitches belonging to the chords. Chord annotations are based on both the melody and the accompaniment.

The comparison of trace prolongations illustrates an important difference in the way chord tones and nonchord tones are used and perceived in tonal melodies, but it does not necessarily show that the prolonged traces contribute to better chord estimation. This is because the above analysis associates the entire length of a trace with the annotated function of the stimulus tone within the chord span in which its note duration falls. It is possible that the oscillatory trace for a chord tone extends into the next chord span where it is not a chord tone, and this would compromise the accuracy of chord estimation. As shown in Figure 4, trace prolongations beyond the current chord span may strengthen or weaken the prominence of chord tones in the next chord span. For example, the trace at E5 starting in the first chord span prolongs into the second span where it remains a chord tone, thereby enhancing the representation of the chord tones. On the other hand, the trace at D5 that begins in the second chord span becomes a nonchord-tone trace in the next span. (It could be argued that this response is not necessarily wrong because the chord annotation is based on both the melody and the accompaniment, while the model is driven by the melody only. It is an empirical question, which is beyond the scope of this study, to what extent the model's response corresponds with the human perception of unaccompanied melodies.)

To investigate the effect of trace prolongation across chord spans, we compared the traces at chord pitches and nonchord pitches within individual chord spans regardless of the origin of the traces. The difference between the total trace durations for chord pitches and the total trace durations for nonchord pitches was taken as the perceptual salience of the annotated chord in the model's response. To evaluate the model's contribution to chord estimation over note durations, the difference in trace duration was then compared to the difference in total note duration between chord tones and nonchord tones in each chord span.

### Methods

The simulation data obtained for Test 1 were used for the analysis of individual chord spans. For each annotated chord span, trace durations and note durations were summed for chord pitches and nonchord pitches separately. The chord boundaries used for calculating trace durations were shifted forward by 40 ms to reflect the typical rise time of Layer 2 oscillations after the stimulus onset. For each chord span, the differences between chord tones and nonchord tones in total trace duration and total note duration were calculated. A *t*-test was performed to determine whether the trace duration differences and the note duration differences are significantly different.

### Results and discussion

**Figure 5:**
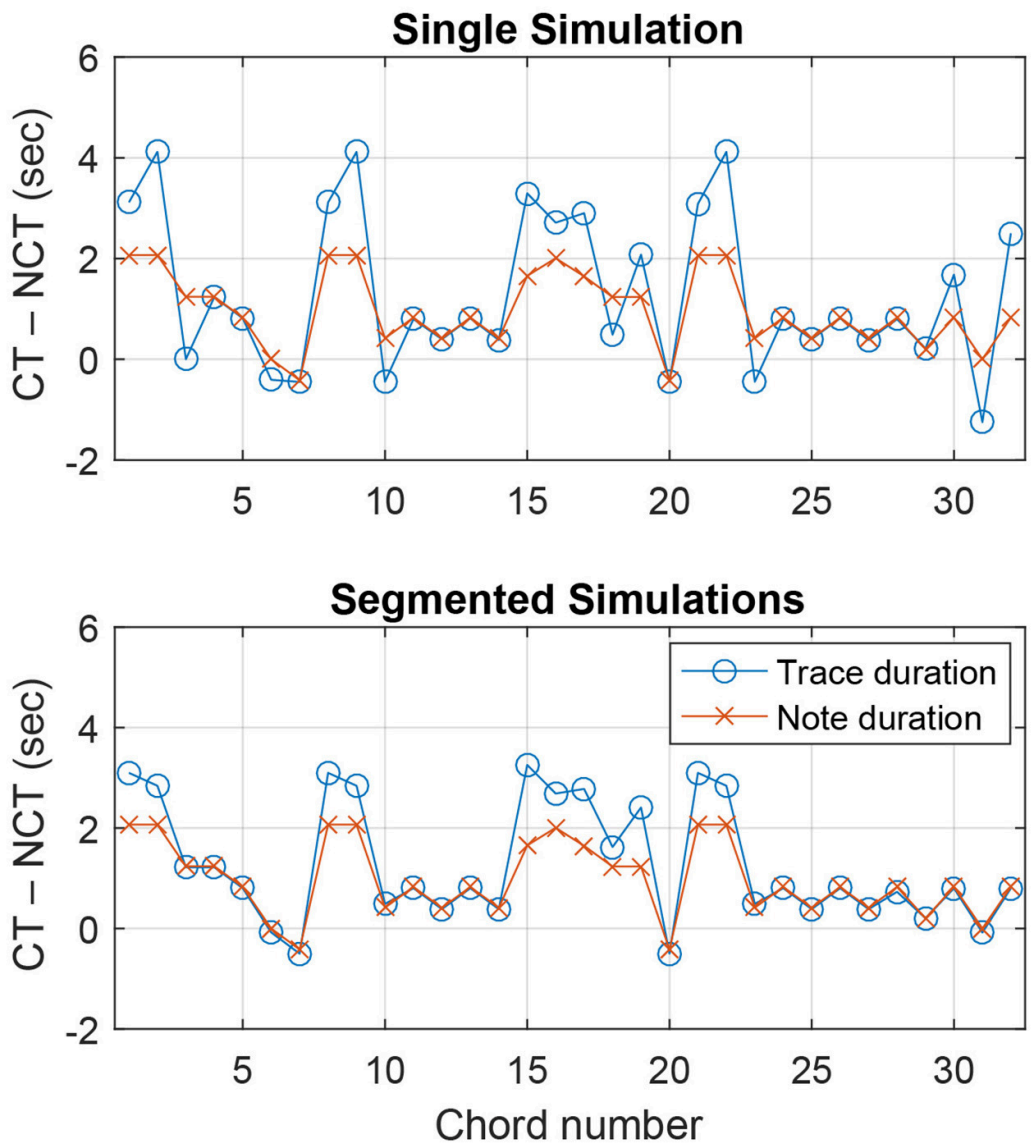
**Difference between chord pitches and nonchord pitches in total trace duration and total note duration within each chord span in Mozart Piano Sonata, K. 331, Theme**. The top panel shows a single simulation run with the entire melody, and the bottom panel shows simulations for individual chord spans run separately. CT and NCT denote chord tones and nonchord tones.

Figure 5 (top) shows the trace duration difference and the note duration difference for each chord span in the theme of K. 331. The graph reflects our observations above. For the second chord span, the trace duration difference is greater than the note duration difference (meaning chord pitches are more emphasized in the model response than in the note durations), while it is the opposite for the third chord span (chord pitches less prominent in the model). For K. 331, the mean trace duration difference between chord pitches and nonchord pitches was 1304 ms, and the mean note duration difference was 973 ms.

**Figure 6:**
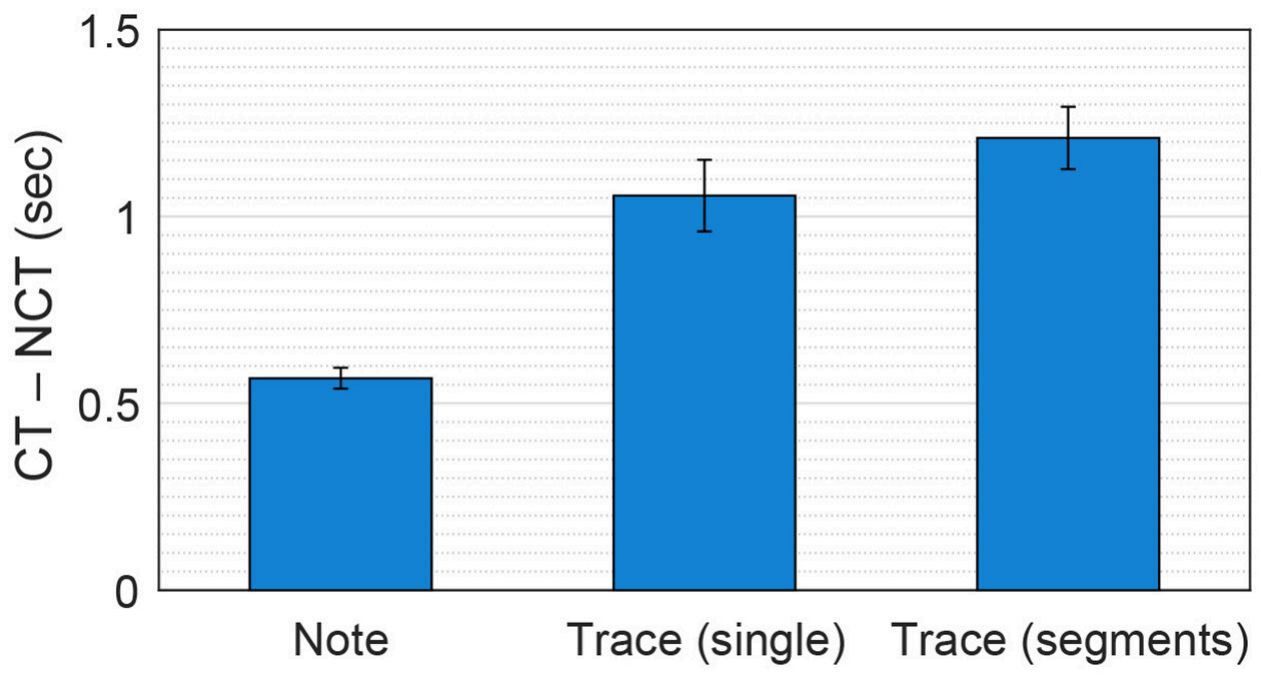
**Mean difference between chord pitches and nonchord pitches in note duration, trace duration in single simulations and trace duration in segmented simulations, averaged over all chord spans in the seven Mozart melodies**. The error bars indicate standard errors. CT and NCT denote chord tones and nonchord tones.

Considering all 405 chord spans in the seven Mozart melodies, trace duration differences and note duration differences were significantly different [paired-sample *t*-test: *t*(404) = 6.21, *p* < 0.001], with the mean values of 1056 ms (trace duration differences) and 567 ms (note duration differences) (see Figure 6). This suggests that, overall, the dynamical model's response can provide a better basis for chord estimation than note durations.

## Test 3: trace durations within segmented chord spans

Despite the overall advantage of trace duration over note duration, there are chord spans for which trace duration performs worse than note duration (see Figure 5, top). As discussed above, the prolongation of pitch traces across chord boundaries could result in less accurate chord representations. This issue points to the importance of segmentation in chord estimation. Previous studies have shown that the accuracy of chord estimation can be improved by synchronizing analysis frames to the beat of the music being analyzed, which tends to align with harmonic changes (Bartsch and Wakefield, 2001; Bello and Pickens, 2005). We tested whether chord estimation based on the pitch memory model could be improved by using segmented stimulus signals. Instead of running the model for entire melodies, we chopped the melodies into individual chord spans and ran the model for each segment separately. This would prevent previous oscillatory traces from extending into the current chord span because each simulation starts anew from small random initial values.

### Methods

A separate stimulus signal was prepared for each chord span in the Mozart melodies (total 405 segments; see General Material and Methods for the general procedures of stimulus preparation), and the model was run for each individual segment separately. As was done for Test 2, the total trace durations and total note durations for chord pitches and nonchord pitches were calculated for each chord span. A *t*-test was performed to determine if trace duration differences and note duration differences are significantly different in segmented chord spans.

### Results and discussion

Figure 5 (bottom) shows trace duration differences and note duration differences for the segmented simulations of K. 331. It can be seen that the trace duration difference is either comparable or greater than the note duration difference for all chord spans. Over all seven melodies, the trace duration differences for segmented simulations (1,211 ms on average) were significantly greater than those for single simulations in Test 2 [*t*(404) = 3.16, *p* < 0.01; see Figure 6]. This shows that, as was found for previous methods using chroma-based features, chord estimation based on the pitch memory model can benefit from processing each chord span separately.

## General discussion

In this paper, we presented a first step toward automatic chord estimation based on nonlinear dynamics, which draws on research in music cognition and auditory neuroscience. As an alternative to the current methods of feature extraction for chord estimation, we used a dynamical model of short-term pitch memory to predict the relative salience of pitches in tonal melodies. We modeled cohesion and competition between melodic pitches as dynamic pattern formation in a gradient frequency neural network, which is a biologically realistic model of auditory neural processing. We tested the model with a collection of unaccompanied melodies and showed that it can provide better mid-level representations for chord estimation than the distribution of note durations which current chroma-based features are aimed to extract from the music signal. It was shown that chord tones are rendered more prominent in the model's response than in the note durations and that the advantage of the model can be increased by using segmented input signals.

The present study is an attempt to bridge music informatics with music cognition by developing a chord estimation method based on the human perception of implied harmony. Much progress has been made in automatic chord estimation, with state-of-the-art systems employing cutting-edge techniques in signal processing and machine learning (see Cho and Bello, 2014; McVicar et al., 2014, for reviews). Recently, however, a plateau in performance was observed despite continuous incorporation of new data-driven methods which have proven to be successful in other machine learning domains (Humphrey and Bello, 2015). This calls for examination of the underlying assumptions of current chord estimation methods and also encourages incorporation of the findings in other related disciplines such as music cognition and auditory neuroscience. Here we showed that the pattern of pitch salience in the dynamical model of auditory short-term memory can provide a better feature for automatic chord estimation than the chroma distribution in the audio signal. The success of the present method demonstrates that human perception and underlying neural mechanisms can provide foundations for breakthroughs in music informatics research. It also warrants further investigation as to whether the dynamical models of auditory neural processing can improve the retrieval of other musical information.

The dynamical model of short-term pitch memory presented in this paper differs from previous models of echoic memory in which individual pitch traces, once initiated, decay monotonically independent of each other (e.g., Huron and Parncutt, 1993; Leman, 2000; Toiviainen and Krumhansl, 2003). In the present model, a pitch trace may sustain for a long time or be suppressed quickly at the offset of the stimulus tone depending on its interaction with other pitch traces, which is consistent with experimental findings on short-term pitch memory (Deutsch, 1972, 1973; Deutsch and Feroe, 1975; Schellenberg and Trehub, 1994, 1996a,b). The pitch dynamics observed in the present model also provides a psychological basis for the music-theoretical concept of *prolongation*, a central principle of the hierarchical organization of tonal music. In Schekerian analysis, prolongation refers to the ways in which a pitch or harmony remains active without physically sounding (Katz, 1935; Forte and Gilbert, 1982; Larson, 1997). The prolongation of pitch traces beyond note durations and the subordination of pitch traces to strong neighbors in the present model correspond directly with the idea of prolongation in music theory.

The dynamical model presented in this paper acts as a feature extractor that provides a novel mid-level representation for chord estimation. Hence, it does not perform chord estimation or labeling by itself. There are multiple ways to use the model for automatic chord estimation. For example, the current methods for estimating chords from feature representations (e.g., template matching and stochastic models) could be applied to the output of the present model. However, our ultimate goal is to expand the current model to perform chord estimation within the dynamical systems framework. This may be done by adding another layer of oscillators that holds information about common chord types by means of long-term Hebbian learning. The present model utilizes short-term plasticity to capture the interaction between pitch traces in short-term auditory memory. Adding long-term plastic connections to the model would lead to pattern formation in two different time scales, and the learning and recognition of common chord types could be modeled in terms of the interaction between layers with plasticity of different time scales.

The introduction of long-term plasticity also means the incorporation of the top-down influence of learned knowledge into the dynamical model. Cognitive psychologists have shown that listeners internalize regularities in tonal music through passive exposure and that the implicit knowledge thus acquired influences subsequent perceptions (Krumhansl, 1990; Tillmann et al., 2000; Pearce and Wiggins, 2012; Rohrmeier and Rebuschat, 2012). The model presented in this paper includes only afferent connections from the stimulus to Layer 1 and then to Layer 2, and the plastic connections adjust quickly to the current states of the oscillators. Thus, the response of the model reflects only the pattern of pitch salience in the short-term context. An extra layer with long-term plastic connections could carry information about frequently encountered chord types beyond the short-term context and modulate the activities in Layer 2 through efferent (top-down) connections. In this way, the influence of both short-term context and long-term knowledge could be accounted for within the dynamical systems framework.

We showed that the prominence of chord tones in the model's response could be raised by using segmented signals. This is because running the model separately for each segment prevents oscillatory traces from intruding into the next segment. The same effect can be achieved by deactivating (or resetting) oscillatory traces at segmentation boundaries while running the model continuously with the entire (unsegmented) signal. Segmentation would benefit chord estimation the most if it aligns with chord span boundaries. Above we used segmentations based on chord annotations, but this information is not available to a system performing automatic chord estimation (actually, that is the information such a system aims to obtain). One possible way to incorporate segmentation into the present model is to couple it with a rhythm model that synchronizes to a musical beat and meter (e.g., Large et al., 2015). In the same spirit as the use of beat-synchronized frames for chroma-based features, the pitch memory model could receive a modulatory signal from the rhythm model which deactivates pitch traces at the time of each downbeat. The pitch memory model, on the other hand, could provide input to the rhythm model at the time of harmonic change, which is an important cue for the perception of rhythm and meter (cf. Papadopoulos and Peeters, 2008).

Here we tested the dynamical model with unaccompanied melodies to focus on the differentiation of chord tones and nonchord tones in the absence of explicit chordal context. We found that the model selectively enhanced chord tones in the melodies, thus raising the probability of correct chord estimation. The results of this study prompt us to ask how well the model would handle music with multiple voices. We predict that the model would still show an advantage over raw pitch-class content. The presence of vertical consonant intervals, which typically form between chord tones, would facilitate the suppression of nonchord tones. Also, we expect the model to capture pitch dynamics within individual voices as it did for single unaccompanied melodies. This prediction will have to be tested in future studies.

## Author contributions

JK designed and ran the model and wrote the paper.

## Funding

This work was supported by NSF BCS-1027761 and AFOSR FA9550-12-10388.

### Conflict of interest statement

The author declares that the research was conducted in the absence of any commercial or financial relationships that could be construed as a potential conflict of interest.

## References

[B1] Apel W. (1969). The Harvard Dictionary of Music, 2nd Edn. Cambridge, MA: Belknap Press.

[B2] Bartsch M. A. Wakefield G. H. (2001). To catch a chorus: using chroma-based representations for audio thumbnailing, in Proceedings of the 2001 IEEE Workshop on the Applications of Signal Processing to Audio and Acoustics (New Paltz, NY: IEEE), 15–18.

[B3] Bello J. P. Pickens J. (2005). A robust mid-level representation for harmonic content in music signals, in Proceedings of the 6th International Conference on Music Information Retrieval (London: Queen Mary, University of London), 304–311.

[B4] Bharucha J. J. (1984). Anchoring effects in music: the resolution of dissonance. Cogn. Psychol. 16, 485–518. 10.1016/0010-0285(84)90018-5

[B5] Bharucha J. J. (1996). Melodic anchoring. Music Percept. 13, 383–400. 10.2307/40286176

[B6] Bingham W. V. D. (1910). Studies in melody. Psychol. Rev. Monogr. Suppl. 12, i–88. 10.1037/h0093021

[B7] Brown H. (1988). The interplay of set content and temporal context in a functional theory of tonality perception. Music Percept. 5, 219–249. 10.2307/40285398

[B8] Camalet S. Duke T. Jülicher F. Prost J. (2000). Auditory sensitivity provided by self-tuned critical oscillations of hair cells. Proc. Natl. Acad. Sci. U.S.A. 97, 3183–3188. 10.1073/pnas.97.7.3183 10737791 PMC16213

[B9] Cho T. Bello J. P. (2014). On the relative importance of individual components of chord recognition systems. IEEE/ACM Trans. Audio Speech Lang. Process. 22, 477–492. 10.1109/TASLP.2013.2295926

[B10] Cuddy L. L. Cohen A. J. Mewhort D. J. K. (1981). Perception of structure in short melodic sequences. J. Exp. Psychol. Hum. Percept. Perform. 7, 869–883. 10.1037/0096-1523.7.4.869 6457099

[B11] Davis S. (2006). Implied polyphony in the solo string works of J. S. Bach: a case for the perceptual relevance of structural expression. Music Percept. 23, 423–446. 10.1525/mp.2006.23.5.423

[B12] de Cheveigné A. (2006). Multiple F0 estimation, in Computational Auditory Scene Analysis: Principles, Algorithms, and Applications, eds Wang D. Brown G. J. (Piscataway, NJ: IEEE Press; Wiley), 45–79.

[B13] Deutsch D. (1972). Mapping of interactions in the pitch memory store. Science 175, 1020–1022. 10.1126/science.175.4025.1020 5009395

[B14] Deutsch D. (1973). Interference in memory between tones adjacent in the musical scale. J. Exp. Psychol. 100, 228–231. 10.1037/h0035440 4745453

[B15] Deutsch D. Feroe J. (1975). Disinhibition in pitch memory. Percept. Psychophys. 17, 320–324. 10.3758/BF03203217

[B16] Erickson R. (1984). A perceptual substrate for tonal centering? Music Percept. 2, 1–5. 10.2307/40285278

[B17] Forte A. Gilbert S. E. (1982). Introduction to Schenkerian Analysis. New York, NY: Norton.

[B18] Fux J. J. (1725). Steps to Parnassus. The Study of Counterpoint. New York, NY: W. W. Norton & Company.

[B19] Holleran S. Jones M. R. Butler D. (1995). Perceiving implied harmony: the influence of melodic and harmonic context. J. Exp. Psychol. Learn. Mem. Cogn. 21, 737–753. 10.1037/0278-7393.21.3.737 7602268

[B20] Hudspeth A. J. Jülicher F. Martin P. (2010). A critique of the critical cochlea: hopf–a bifurcation–is better than none. J. Neurophysiol. 104, 1219–1229. 10.1152/jn.00437.2010 20538769 PMC2944685

[B21] Humphrey E. J. Bello J. P. (2015). Four timely insights on automatic chord estimation, in Proceedings of the 16th International Society for Music Information Retrieval Conference (Málaga), 673–679.

[B22] Huron D. Parncutt R. (1993). An improved model of tonality perception incorporating pitch salience and echoic memory. Psychomusicology 12, 154–171. 10.1037/h0094110

[B23] Jiang N. Grosche P. Konz V. Müller M. (2011). Analyzing chroma feature types for automated chord recognition, in Audio Engineering Society Conference: 42nd International Conference: Semantic Audio (Ilmenau).

[B24] Katz A. T. (1935). Heinrich Schenker's method of analysis. Music. Q. XXI, 311–329. 10.1093/mq/XXI.3.311

[B25] Kim J. C. (2011). Tonality in Music Arises from Perceptual Organization. Unpublished doctoral dissertation, Northwestern University.

[B26] Kim J. C. Large E. W. (2015). Signal processing in periodically forced gradient frequency neural networks. Front. Comput. Neurosci. 9:152. 10.3389/fncom.2015.00152 26733858 PMC4689852

[B27] Krumhansl C. L. (1990). Cognitive Foundations of Musical Pitch. New York, NY: Oxford University Press.

[B28] Krumhansl C. L. Cuddy L. L. (2010). A theory of tonal hierarchies in music, in Music Perception, Vol. 36, eds Riess Jones M. Fay R. R. Popper A. N. (New York, NY: Springer), 51–87.

[B29] Large E. W. Almonte F. V. Velasco M. J. (2010). A canonical model for gradient frequency neural networks. Phys. D Nonl. Phenom. 239, 905–911. 10.1016/j.physd.2009.11.015

[B30] Large E. W. Herrera J. A. Velasco M. J. (2015). Neural networks for beat perception in musical rhythm. Front. Syst. Neurosci. 9:159. 10.3389/fnsys.2015.00159 26635549 PMC4658578

[B31] Large E. W. Kim J. C. Flaig N. K. Bharucha J. J. Krumhansl C. L. (2016). A neurodynamic account of musical tonality. Music Percept. 33, 319–331. 10.1525/mp.2016.33.3.319

[B32] Large E. W. Kim J. C. Lerud K. D. Harrell D. (2014). GrFNN Toolbox: Matlab Tools for Simulating Signal Processing, Plasticity and Pattern Formation in Gradient Frequency Neural Networks. Available online at: https://github.com/MusicDynamicsLab/GrFNNToolbox

[B33] Large E. W. Kozloski J. R. Crawford J. D. (1998). A dynamical model of temporal processing in the fish auditory system, in Association for Research in Otolaryngology Abstracts Vol. 21. (St. Petersburg, FL), 717.

[B34] Larson S. (1997). The problem of prolongation in tonal music: terminology, perception, and expressive meaning. J. Music Theor. 41, 101–136. 10.2307/843763

[B35] Laudanski J. Coombes S. Palmer A. R. Sumner C. J. (2010). Mode-locked spike trains in responses of ventral cochlear nucleus chopper and onset neurons to periodic stimuli. J. Neurophysiol. 103, 1226–1237. 10.1152/jn.00070.2009 20042702 PMC2887620

[B36] Lee K. Slaney M. (2006). Automatic chord recognition from audio using a supervised HMM trained with audio-from-symbolic data, in AMCMM '06 Proceedings of the 1st ACM Workshop on Audio and Music Computing Multimedia (Santa Barbara, CA: ACM Press), 11–20.

[B37] Leman M. (2000). An auditory model of the role of short-term memory in probe-tone ratings. Music Percept. 17, 481–509. 10.2307/40285830

[B38] Lerud K. D. Almonte F. V. Kim J. C. Large E. W. (2014). Mode-locking neurodynamics predict human auditory brainstem responses to musical intervals. Hear. Res. 308, 41–49. 10.1016/j.heares.2013.09.010 24091182

[B39] Lerud K. D. Kim J. C. Almonte F. V. Carney L. H. Large E. W. (2015). A canonical nonlinear cochlear model, in Association for Research in Otolaryngology Abstract, Vol. 38 (Baltimore, MD), 211–212.

[B40] McVicar M. Santos-Rodriguez R. Ni Y. Bie T. D. (2014). Automatic chord estimation from audio: a review of the state of the art. IEEE/ACM Trans. Audio Speech Lang. Process. 22, 556–575. 10.1109/TASLP.2013.2294580

[B41] Papadopoulos H. Peeters G. (2008). Simultaneous estimation of chord progression and downbeats from an audio file, in IEEE International Conference on Acoustics, Speech, and Signal Processing (Las Vegas, NV: IEEE), 121–124.

[B42] Pardo B. Birmingham W. P. (2002). Algorithms for chordal analysis. Comput. Music J. 26, 27–49. 10.1162/014892602760137167

[B43] Pearce M. T. Wiggins G. A. (2012). Auditory expectation: the information dynamics of music perception and cognition. Top. Cogn. Sci. 4, 625–652. 10.1111/j.1756-8765.2012.01214.x 22847872

[B44] Povel D.-J. Jansen E. (2002). Harmonic factors in the perception of tonal melodies. Music Percept. 20, 51–85. 10.1525/mp.2002.20.1.51

[B45] Rohrmeier M. Rebuschat P. (2012). Implicit learning and acquisition of music. Top. Cogn. Sci. 4, 525–553. 10.1111/j.1756-8765.2012.01223.x 23060126

[B46] Schellenberg E. G. Trehub S. E. (1994). Frequency ratios and the discrimination of pure tone sequences. Percept. Psychophys. 56, 472–478. 10.3758/BF03206738 7984402

[B47] Schellenberg E. G. Trehub S. E. (1996a). Children's discrimination of melodic intervals. Dev. Psychol. 32, 1039–1050. 10.1037/0012-1649.32.6.1039

[B48] Schellenberg E. G. Trehub S. E. (1996b). Natural musical intervals: evidence from infant listeners. Psychol. Sci. 7, 272–277. 10.1111/j.1467-9280.1996.tb00373.x

[B49] Schenker H. (1956). Free Composition: Volume III of New Musical Theories and Fantasies, 2nd Edn. Longman music series. New York, NY: Longman.

[B50] Tan N. Aiello R. Bever T. G. (1981). Harmonic structure as a determinant of melodic organization. Mem. Cogn. 9, 533–539. 10.3758/BF03202347 7321871

[B51] Temperley D. (2007). The melodic-harmonic ‘divorce’ in rock. Popular Music 26, 323–342. 10.1017/S0261143007001249

[B52] Thomson W. (1999). Tonality in Music: A General Theory. San Marino, CA: Everett Books.

[B53] Tillmann B. Bharucha J. J. Bigand E. (2000). Implicit learning of tonality: a self-organizing approach. Psychol. Rev. 107, 885–913. 10.1037/0033-295X.107.4.885 11089410

[B54] Toiviainen P. Krumhansl C. L. (2003). Measuring and modeling real-time responses to music: the dynamics of tonality induction. Perception 32, 741–766. 10.1068/p3312 12892434

[B55] Trainor L. J. Trehub S. E. (1994). Key membership and implied harmony in Western tonal music: Developmental perspectives. Percept. Psychophys. 56, 125–132. 10.3758/BF03213891 7971113

[B56] Zarlino G. (1558). The Art of Counterpoint. Part Three of Le Istitutioni Harmoniche. Music theory translation series. New Haven, CT: Yale University Press.

